# Supplementary material for: Similar connectivity of gut microbiota and brain activity networks is mediated by animal protein and lipid intake in children from a Mexican indigenous population
Source: PLoS One. 2023 Jun 29;18(6):e0281385. doi: 10.1371/journal.pone.0281385 (PMC10310019; doi:10.1371/journal.pone.0281385)
Supplement: S1 File — (PDF) [file pone.0281385.s001.pdf]

Supplementary materials: Diet influenceSimilar connectivity of gut microbiota and brain activity networks is mediated by animal protein and lipid intake in children from a Mexican indigenous population..

Elvia Ramírez-Carrillo[1 *], Isaac G-Santoyo[1,2*], Oliver López-Corona[3,4*], Luisa I. Falcón[6], Osiris Gaona[6], Daniel Cerqueda-García[7], Andrés Sánchez-Quinto[6], Olga A. Rojas-Ramos[1,5], Rosa María de la Fuente Rodríguez[1], Ariatna Hernández Castillo[1], Diego Hernández-Muciño[8], and Javier Nieto[9]

*elviarc@otrasenda.org
*isantoyo@psicologia.unam.mx
*lopezoliverx@ciencias.unam.mx

[1]NeuroEcology Lab, Department of Psychology, UNAM, México, 04510
[2]Unidad de Investigación en Psicobiologia y Neurociencias, Department of Psychology, UNAM, México, 04510
[3]Cátedras CONACyT, Comisión Nacional para el Conocimiento y Uso de la Biodiversidad (CONABIO), CDMX, México
[4]Centro de Ciencias de la Complejidad (C3), Universidad Nacional Autónoma de México, CDMX, México
[5]Coordinación de Psciobiología y Neurociencias, Facultad de Psicología, UNAM, México, 04510
[6]Laboratorio de Ecología Bacteriana, Instituto de Ecología, Universidad Nacional Autónoma de México, UNAM, Parque Científico y Tecnológico de Yucatán, Mérida, México, 97302.
[7]Consorcio de Investigación del Golfo de México (CIGoM), Centro de Investigación y de Estudios Avanzados del Instituto Politécnico Nacional, Unidad Mérida, Departamento de Recursos del Mar, Mérida, Yucatán, México.
[8]Laboratorio de Agroecología Instituto de Investigaciones en Ecosistema y Sustentabilidad, UNAM, Morelia, México
[9]Laboratorio de Aprendizaje y Adaptación, Facultad de Psicología, UNAM, México, 04510

Supplementary Materials

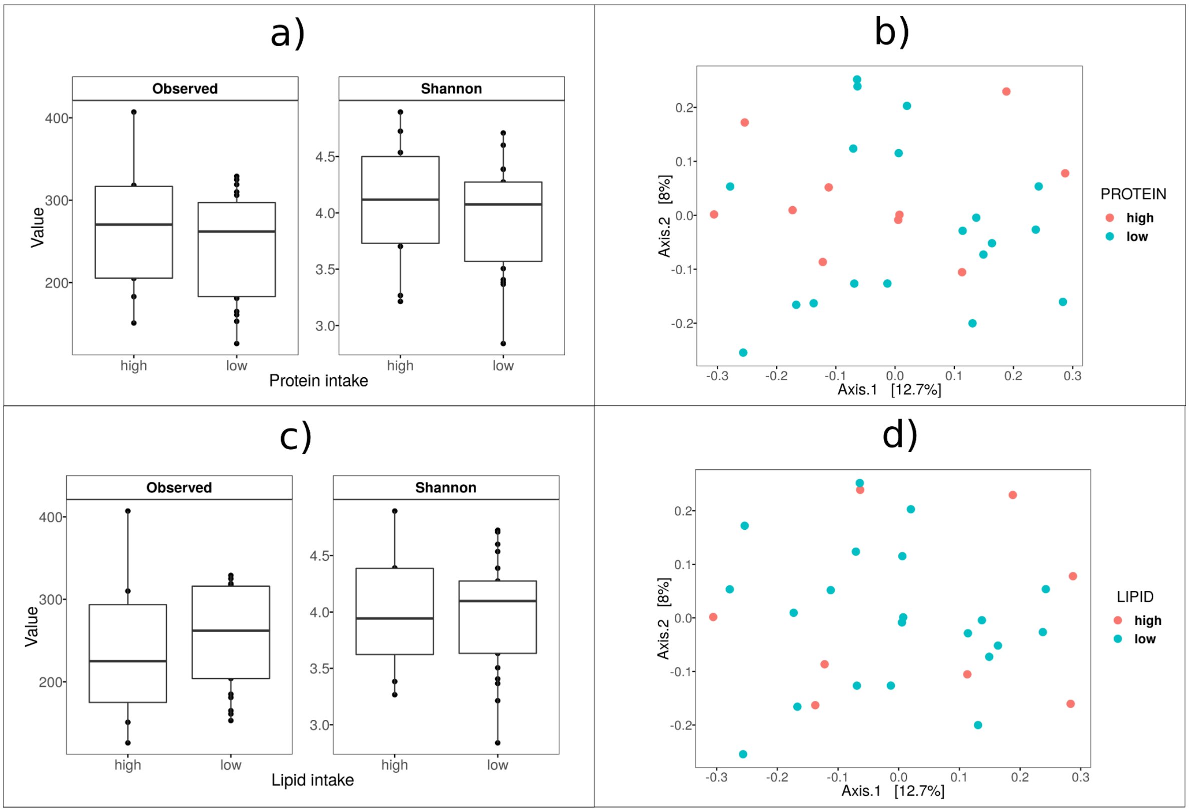

Sup.Fig1. Alpha diversity indexes and Beta ordinations of children GM by protein and lipid groups consumption. The boxplot with a median of Observed ASVs and Shannon index for protein (a) and lipids (c). (b) Corresponds to Unweighted Unifrac for proteins and (d) for lipids. There were no statistical differences in any diversity index (alpha and beta) between low and high consumption groups

a)

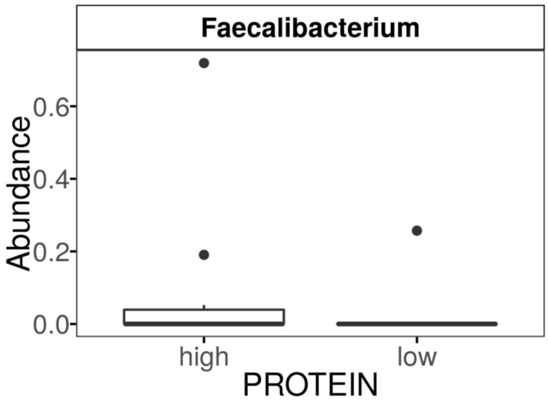

b)

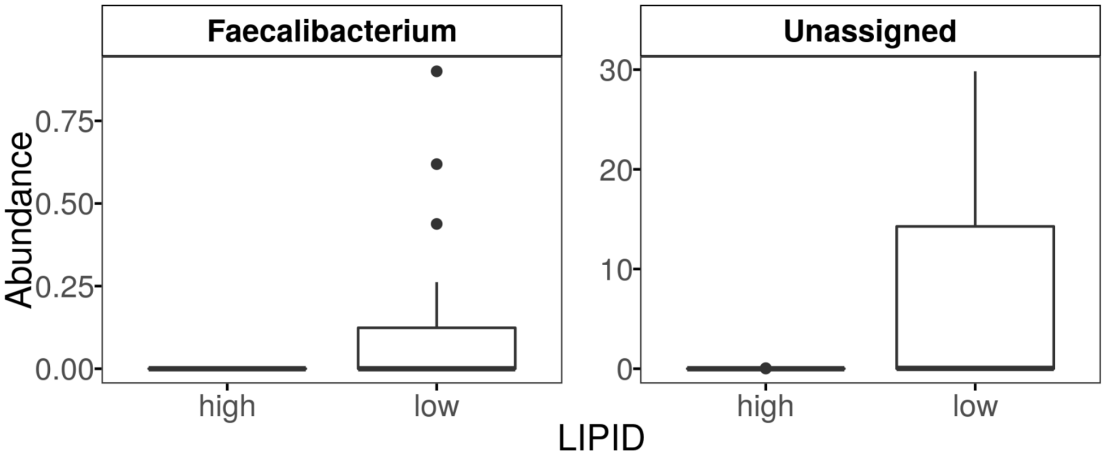

Sup.Fig2. Log2 fold-change analysis of the combined children GM between Low and High proteins (a) and lipids (b)consumption. Fecal-prokaryotic ASVs are grouped by genus. The figure shows the relative abundance of each discriminant group. Genus observed in this figure were statistically significantly different between Low and High consumption at $p < 0.01$ corrected with the FDR (false discovery rate) method.
