# Supplementary material for: Similar connectivity of gut microbiota and brain activity networks is mediated by animal protein and lipid intake in children from a Mexican indigenous population
Source: PLoS One. 2023 Jun 29;18(6):e0281385. doi: 10.1371/journal.pone.0281385 (PMC10310019; doi:10.1371/journal.pone.0281385)
Supplement: S2 File — (PDF) [file pone.0281385.s002.pdf]

## ANEXO II. CUESTIONARIO DE FRECUENCIA DE CONSUMO DE ALIMENTOS

**NOMBRE____________________________________________________________________.**
**NOMBRE DE LA MADRE _______________________________________________________ .**
**CÓDIGO______________________________.**
**FECHA_______________________________.**

**INSTRUCCIONES:** A continuación, se presenta una lista de alimentos, de las cuales usted deberá colocar en la casilla correspondiente la frecuencia de veces a la semana y veces al día en que consume su hijo consume cada uno de los alimentos, tomando en cuenta la porción que se especifica.

**INSTRUCCIONES PARA EL APLICADOR:** Si usted aplicará el cuestionario a otra persona, debe hacer la pregunta ¿Cuántos días a la semana su hijo come o toma (la porción del alimento especificada)? ¿Cuántas veces al día? Y colocar la respuesta en la casilla correspondiente.

| Alimento | Porción | Días de la semana ¿Cuántos días comió o tomó usted? | Veces al día ¿Cuántas veces al día comió o tomó usted? |
|---|---|---|---|
| Leche | 1 vaso (240ml) | | |
| Yogur | 1 envase (150g) | | |
| Naranja o mandarina | 1 pieza mediana | | |
| Plátano | 1 pieza mediana | | |
| Manzana o pera | 1 pieza mediana | | |
| Melón o sandía | 1 pieza mediana | | |
| Guayaba | 1 pieza mediana | | |
| Papaya | 1 rebanada mediana | | |
| Fresa | 1 taza (140g) | | |
| Jitomate | ½ pieza chica (30g) | | |
| Hojas verdes (acelgas, espinacas, quelites) | ½ plato (85 g) cocidas o 1 plato crudas | | |
| Aguacate | 1 rebanada (33g) | | |
| Zanahoria | 1 pieza chica (50g) | | |
| Brócoli | ¼ taza | | |
| Elote | ½ pieza chica (50g) | | |
| Nopales | 1 pieza mediana (70g) | | |
| Chile | 1 pieza mediana (80g) | | |
| Cebolla | 1 cucharada sopera (7g) | | |
| Carne de puerco | 1 bistec chico (55g) | | |
| Carne de res | 1 bistec chico (55g) | | |
| Pollo | 1 pieza (90g) | | |
| Huevo | 1 pieza entera (62g) | | |
| Pescado | ½ filete mediano (45g) | | |
| Frijoles de la olla | ½ plato (50g) | | |
| Lenteja, garbanzo, haba, alubias | ½ plato (50g) | | |
| Arroz | 1 taza (100g) | | |

| | | | |
|---|---|---|---|
| Papa | ½ taza mediana cocida (40g) | | |
| Tamal | 1 pieza (200g) | | |
| Atole de maíz | 1 taza (240ml) | | |
| Refresco | 1 vaso (240ml) | | |
| Jugos naturales | 1 vaso (240ml) | | |
| Dulces | 1 pieza (30g) | | |
| Frituras | 1 paquete individual (35g) | | |
| Caldo de pollo | ½ taza (120ml) | | |
| Azúcar | 1 cucharada sopera (10g) | | |
| Manteca animal | 1 cucharada sopera (10g) | | |
| Sal | | | |
| Tortilla (gr) | | | |
| Tortilla hecha en casa (gr) | | | |

**INSTRUCCIONES:** Responda las siguientes preguntas acerca de su embarazo y primeros años de vida de su hijo o hija.

¿Hay algún alimento que usted consuma con frecuencia y no se encuentre en la lista? Especifique el alimento y la porción que consume.

_______________________________________.

¿Cuántas veces a la semana lo consume?_________________________________.

¿Cuántas veces al día?____________________________________.

Tiempo de lactancia (especifique meses o años)___________________________________.

Tiempo de gestación (meses)__________________________________.

Tipo de nacimiento_________________________________________.

¿Consumió algún suplemento durante el embarazo?_______________________________.

Especifique la cantidad y frecuencia_____________________________________________.

¿Su hijo consumió algún suplemento alimenticio durante los primeros años de vida?____________________________.
