# Supplementary material for: Similar connectivity of gut microbiota and brain activity networks is mediated by animal protein and lipid intake in children from a Mexican indigenous population
Source: PLoS One. 2023 Jun 29;18(6):e0281385. doi: 10.1371/journal.pone.0281385 (PMC10310019; doi:10.1371/journal.pone.0281385)
Supplement: S3 File — (PDF) [file pone.0281385.s003.pdf]

# Food Frequency Intake

**Name**_______________________________________________________________.
**Mother's Name** _________________________________________________________.
**Code**_____________________________.
**Date**______________________________.

**General Instructions:** At the food list as follows, please write down the frequency of consumption of each element by week and by day. Take in consideration the specific portion of each food.

**Applicator Instructions:** If you are going to fill the questionnaire, ask: How many days a week do your child consume (the specified portion of each food)? How many times in a day? Write down the answer at the corresponding space.

| Food | Portion | Days in a week | Times in a day |
|---|---|---|---|
| Milk | 1 glass (8 oz) | | |
| Yogurt | 5 oz | | |
| Orange or tangerines | 1 medium piece | | |
| Banana | 1 medium piece | | |
| Apple or pear | 1 medium piece | | |
| Watermelon or cantaloupe | 1 medium piece | | |
| Papaya | 1 medium slice | | |
| Strawberry | 1 cup (5 oz) | | |
| Tomato | ½ of a small piece | | |
| Green leaves (spinach, chard, quelites) | ½ cooked plate (85 g) or 1 raw plate | | |
| Avocado | 1 slice (33 g) | | |
| Carrot | 1 small piece | | |
| Broccoli | ¼ cup | | |
| Corn | ½ small piece (50g) | | |

| | | | |
|---|---|---|---|
| Nopales | 1 medium piece (70g) | | |
| Chili | 1 medium piece (80 g) | | |
| Onion | 1 tablespoon (7 g) | | |
| Pork meat | 1 small steak (55 g) | | |
| Beef | 1 small steak (55 g) | | |
| Chicken | 1 piece ( 90 g) | | |
| Egg | 1 piece (62 g) | | |
| Fish | ½ medium steak (45 g) | | |
| Beans | ½ plate (50 g) | | |
| Lentil, chickpea, broad bean | ½ plate (50 g) | | |
| Rice | 1 cup ( 100 g) | | |
| Potato | ½ medium cooked cup | | |
| Tamal | 1 piece (200 g) | | |
| Corn Atole | 1 cup (8 oz) | | |
| Soda | 1 glass (8 oz) | | |
| Natural Juice | 1 glass (8 oz) | | |
| Candies | 1 piece (30 g) | | |
| Potato Chips | 1 individual package (35 g) | | |
| Chicken soup | ½ cup (4 oz) | | |
| Sugar | 1 tablespoon | | |
| Butter | 1 tablespoon | | |
| Tortilla | 1 piece | | |
| Homemade Tortilla | 1 piece | | |

Do you consume another food regularly that is not on the list? Specify the food and the portion of it.

________________________________________________.

How many times a week and a day do you consume it?

________________________________________________.

**Instructions:** Answer the next questions about your pregnancy and your child's first years of life.

For how long did you breastfeed your child? (months, years) _____________________.

How many months did your pregnancy take?_________________.

What type of delivery did you have? (vaginal delivery or cesarean section) ________________.

Did you take supplements during your pregnancy?____________________.
Specify the quantity and frequency of consumption____________________.

Did your child consume nutritional supplements in his/hers first two years of life? __________________________.
