# Supplementary material for: Similar connectivity of gut microbiota and brain activity networks is mediated by animal protein and lipid intake in children from a Mexican indigenous population
Source: PLoS One. 2023 Jun 29;18(6):e0281385. doi: 10.1371/journal.pone.0281385 (PMC10310019; doi:10.1371/journal.pone.0281385)
Supplement: S4 File — (PDF) [file pone.0281385.s004.pdf]

Inclusivity in global research

PLOS' policy on inclusivity in global research aims to improve transparency in the reporting of research performed outside of researchers' own country or community and ensures that PLOS publications reporting global research adhere to high standards for research ethics and authorship. Authors of relevant research articles may be asked to complete the questionnaire below, which outlines ethical, cultural, and scientific considerations specific to inclusivity in global research. This questionnaire may be requested when researchers have travelled to a different country to conduct research, if research uses samples collected in another country, research with Indigenous populations or their lands, or if research is on cultural artefacts. Researchers travelling to another country solely to use laboratory equipment will not normally be required to complete the questionnaire. However, the questionnaire can be requested at the journal's discretion for any submission – if you have been requested to complete this questionnaire by the PLOS journal you submitted to, please do so.

Please complete the questionnaire below and include this as a Supporting Information file with your manuscript. Note that if your paper is accepted for publication, this checklist will be published with your article in the supporting information files. Please ensure that you reference the checklist in the main body of your manuscript. We suggest adding a subsection 'Inclusivity in global research' to your Methods section and adding the following sentence: "Additional information regarding the ethical, cultural, and scientific considerations specific to inclusivity in global research is included in the Supporting Information (SX Checklist)"

The questions have been designed to be applicable to a wide range of study types, and there are subsections for both human subjects research and non-human subjects research. If any of the questions are not relevant to your research please mark them as "N/A" as appropriate.

Ethical considerations, permits and authorship

This section is applicable to all research types.

RProvide details as to who granted permissions and/or consent for the study to take place in the Methods section of your manuscript. This should include the names of **all** ethics boards, governmental organizations, community leaders or other bodies that provided approval for the study. If individuals provided approval refer to these people by their role or title but do not list their name(s).
Response:

The first section of the methods includes all requested information

If there were any deviations from the study protocol after approval was obtained please provide details of these changes in the Methods section of your manuscript.

Response:

N/A

Did this study involve local collaborators that are residents of the country where the research was conducted or members of the community studied? If you do not have any authors from said communities, please provide an explanation for this below.

Response:

Yes, the study involves local collaborators that are residents of the communities where we performed the research. In particular, we collaborated with a no governmental organization (NGO) named Xuajin Me'phaa, which comprises members of these communities and promotes the region's social, environmental, and economic development ((see video from this organisation, https://youtu.be/WOEcGUHjR9Q). XuaJin Me'phaa was the liaison between communities and our research group, who also helped us by supplying two experienced interpreters of Me'Phaa language, Julio Santiago Naranjo and Ines Guzman. Both are full-time workers in this NGO and participated in all meetings between community members and our research group. Moreover, Diego Hernandez-Muciño, co-author of this manuscript, is the external academic consultant and academic research manager of this NGO. Additionally, we explicitly acknowledge this NGO, her chief executive officer, margarita muciño, and the two interpreters mentioned above. All this information is included in the acknowledges section, and the methods section, specifically in the subsection to recruit participants.

Everyone listed as an author should meet PLOS' criteria for authorship and all individuals who meet these criteria should be included in the author byline, rather than the acknowledgements. Authorship criteria is based on the International Committee of Medical Journal Editors (ICMJE) Uniform Requirements for Manuscripts Submitted to Biomedical Journals - for further information please see here: https://journals.plos.org/plosone/s/authorship.

Human subjects research (e.g. health research, medical research, cross-cultural psychology)

Did you obtain written informed consent from a representative of the local community or region before the research took place? How did you establish who speaks for the community? Details of written informed consent obtained from study participants should be reported separately in the Methods section of your manuscript.

Response:

We obtained an informed consent letter from the local community representative through the Xuajin Mephaa NGO. The local representative, named "Tunga", is elected annually by the community and is empowered to establish bridges of dialogue between the community and visitors, according to their regulations on uses and customs. All this information is found in the procedures to recruit participants section.

How did members of the local community provide input on the aims of the research investigation, its methodology, and its anticipated outcome(s)?

Response.

The communities received all research information through assemblies organized by the NGO Xuajin Me'Phaa and the local authority "Tunga." The two interpreters and the "Tunga" translated these assemblies. As was metinoned previously, interpreters belong to this NGO and are members of the same community.

When engaging with the local community, how did you ensure that the informed consent documents and other materials could be understood by local stakeholders?

Response.

All documents that participants signed were translated from Spanish into the Me'phaa language by the two interpreters trained by the NGO. Similarly, questionnaires were translated, applied, and validated by the interpreters, a council of the NGO, and the "Tunga" as the local representative.

Will the findings of the research be made available in an understandable format to stakeholders in the community where the study was conducted (e.g. via a presentation, summary report, copies of publications, etc.)? Please provide details of how this will be achieved.

We provided the following ways of communication with communities to report the results we obtained.

1) We held three informative Assemblies in each community, preceded by the "Tunga", the interpreters, and the representatives of the NGO.

2) We provided a detailed report in the Mepha language to each participant, explaining the meaning of each of the variables obtained in the study.

3) We performed two assemblies with higher government authorities to explain the study results with the aim of complementing the social development plans of the communities.

The mentioned avenues for informing have been successful in previous studies, allowing us to participate actively in these communities over time. The success of these strategies is observable through different works published. Below are some examples:

Miramontes, O., DeSouza, O., Hernández, D. & Ceccon, E. Non-Lévy Mobility Patterns of Mexican Me’Phaa Peasants Searching for Fuel Wood. Hum. Ecol. 40, 167–174 (2012).

Hernández-Muciño, D. et al. La comunidad me’phaa construye su futuro: agroecología y restauración como herramientas de desarrollo rural sustentable. in Experiencias de colaboración transdisciplinaria para la sustentabilidad (eds. Merçon, J., Ayala- Orozco, B. & Rosell, J. A.) 66–79 (CopIt ArXives, 2018).

Leongoméz, J. D., Sánchez, O. R., Vásquez-Amézquita, M., Valderrama, E., Castellanos-Chacón, A., Morales-Sánchez, L., ... & González-Santoyo, I. (2020). Self-reported health is related to body Height and waist circumference in rural indigenous and urbanised Latin-American populations. Scientific reports, 10(1), 1-13.

Sánchez-Quinto, A., Cerqueda-García, D., Falcón, L. I., Gaona, O., Martínez-Correa, S., Nieto, J., & G-Santoyo, I. (2020). Gut microbiome in children from indigenous and urban communities in México: Different subsistence models, different microbiomes. Microorganisms, 8(10), 1592.

Ramírez-Carrillo, E., Gaona, O., Nieto, J., Sánchez-Quinto, A., Cerqueda-García, D., Falcón, L. I., ... & González-Santoyo, I. (2020). Disturbance in human gut microbiota networks by parasites and its implications in the incidence of depression. *Scientific Reports*, *10*(1), 1-12.

Non-human subjects research using specimens/ animals collected as part of the study, or those housed in archival collections. Examples include archaeology, paleontology, botany and zoology.

Did the permission you obtained from a local authority to perform the study include an agreement on access to outputs and benefit sharing? This may include procedures to enable fair distribution of the benefits and resources arising from the research performed. Please include any details of Prior Informed Consent and Benefit Sharing Agreements obtained. These may be required by field-specific regulations, for example the Convention on Biological Diversity (CBD) and the associated Nagoya Protocol.

N/A

N/A

If the material used in your study was imported, please A) provide the year it was imported and B) indicate whether permits were obtained to import/export the materials used, C) provide details of any permits obtained. If this information is not available, please indicate this.

If you used archival specimens, please state how the material used in your study was acquired by the institute it is held in and provide details of any permits obtained for the original excavations/ sample collection. If this information is not available, please indicate this.

N/A

How was the potential cultural significance of the materials collected in your study to local communities considered in your research design? Were Indigenous peoples and/or local researchers and institutions involved with archaeological excavations / collection of specimens? If so, please provide a description of their involvement.

N/A

If your manuscript includes photographs of human remains please indicate whether authors obtained permission from descendants or affiliated cultural communities to do so.

N/A
