# Supplementary material for: Similar connectivity of gut microbiota and brain activity networks is mediated by animal protein and lipid intake in children from a Mexican indigenous population
Source: PLoS One. 2023 Jun 29;18(6):e0281385. doi: 10.1371/journal.pone.0281385 (PMC10310019; doi:10.1371/journal.pone.0281385)
Supplement: Attachment — Submitted filename: Response_letter.pdf [file pone.0281385.s005.pdf]

Response Letter Major Rev. 1
PONE-D-22-17440

Similar connectivity of gut microbiota and brain activity networks is mediated by animal protein and lipid intake in children from a Mexican indigenous population.

We are thankful for useful comments by reviewers that have improved the quality of the work. Here we track how we took editor and reviewers comments into account.

PLoS comments	
Comment	Response
In addition, attention must be paid to several details (example: page 4, 33 children are mentioned, divided into 12 males and 19 females. But 19 + 12 = 31)	We revised and corrected, 31 is the correct number of participants. Thank you for noticing it.
Please ensure that your manuscript meets PLOS ONE's style requirements, including those for file naming. The PLOS ONE style templates can be found at https://journals.plos.org/plosone/s/file?id=wjVg/PLOSOne_formatting_sample_main_body.pdf and https://journals.plos.org/plosone/s/file?id=ba62/PLOSOne_formatting_sample_title_authors_affiliations.pdf	We revised this with as much detail as we could
Please include a complete copy of PLOS' questionnaire on inclusivity in global research in your revised manuscript. https://journals.plos.org/plosone/s/best-practices-in-research-reporting. Please upload a completed version of your questionnaire as Supporting Information when you resubmit your manuscript.	Included now as Supporting Information file
Provide as a Supporting Information file the questionnaire used in this study (Spanish and Englis)	Included now as Supporting Information file
A copy of your manuscript showing your	We include in the re-submission a highlighted

changes by either highlighting them or using track changes (uploaded as a *supporting information* file)	PDF with changes.
Please update your submission to use the PLOS LaTeX template	We move our manuscript from MSWord to LaTex using the PLoS-One Template
Include this paragraph in the Acknowledgments Section of your manuscript:<br><br>"We are grateful to Margarita Muciño, Julio Naranjo from Xuajin Me'Pha a non-governmental association, for their help in the liaison with the Me'Phaa community, their help in sample collection and logistics. We also are grateful to Santiago Martinez-Correa for their contribution in DNA amplification. IG-S explicitly acknowledges the UNAM and PASPA-DGAPA program for the financial support granted to carry out his sabbatical stay and the development of this project"	Thank you it is now included
Remove funding information from MS and if needed request update from funding statement, currently: "This project was funded by UNAM-PAPIIT [grant numbers IA209416, IA207019], DGAPA postdoctoral Fellowship, CONACYT Ciencia Básica [grant number 241744] and Instituto de Ecología-UNAM [L.I.F]."	Thank you for noticing this format inconsistency, we removed it and followed subsequent instructions as close as we could.
Thank you for submitting the above manuscript to PLOS ONE. During our internal evaluation of the manuscript, we found significant text overlap between your submission and the following previously published work of which you are an author.<br><br>- https://www.biorxiv.org/content/10.1101/2020.07.25.221408v1	This is not a published previous work. It is a prerpint at bioarxiv on this same work, so no overlap text at all. We changed the title and some parts of the MS to better highlight the main idea, but it is not a separate work and does not constitute any type of overlapping nor self-plagiarism.
Must be "protein and lipid intake" (singular), not protein**s** and lipid**s** intake. Please change it throughout the manuscript.	Thank you for pointing this out, we revised the whole document for this and changed it.

Be consistent with how protein and lipid are written. It must be lower case, but sometimes they are written as Protein and Lipid	
GM is "gut microbiota". Why call them "gut bacteria microbiota"? (See Abstract). Bacteria are part of the microbiota, no need of repeating "bacteria". Moreover, it could be gut microbiota bacteria, not gut bacteria microbiota (which makes no sense). Also, bacteria are plural, so it's bacteria ARE/WERE, not bacteria IS/WAS (see page 5).	Thank you for the suggestion, it is now corrected
Page 13: Must be: This kind of population differS (third person singular)	Thank you, we corrected it.
Page 14: "but they are also present notably differences in factors...". Please rephrase. The sentence is not correct. Perhaps THERE ARE also differences?	Thank you for pointing this out, we rephrased it.
Reviewer #1	
The author compared weight of the MST between high/low protein or lipid intake, showed the difference in percentage. But there is no statistic test to prove this difference is due to the high/low protein or lipid intake. In other words, how the difference of weight of the MST would be, if randomly divide the data in two groups?<br><br>The strategy to divide the children into two group based on mean proteins/lipids intake is not a proper way. For instance, for a normal distribution data, the data points are centered around mean. Arbitrarily assign those data to high or low won't change the fact that they are close; for a u-shaped distribution, the major data points are separated, and far from mean, it's proper to separate them by mean. Back to this study, it seems for protein intake, there are two peaks in distribution; but for lipid intake, the high and low are very close. It would be better to separate the lipid intake into three groups divided by the percentiles.	This question about MST and randomly dividing the data into two groups, is a very insightful question. Let first consider that If there were no underlying ecological (external to the individual) population process, the mutual information matrix (MI) would be very homogeneous. These coefficients of the MI matrix are used as the weight of the networks, so weights are also very homogeneous. If we randomly construct k subnets of sizes n, m (with n<m); and then compare pairs of subnets of n nodes vs. subnets of m nodes, as the weights are very similar; then the value of the MST will change mainly in function of the size of the network. This would mean that the MST of networks of m nodes would be greater than the MST of n nodes. In the case of studies, the subnetwork with the highest number of nodes corresponds to the lowest intake of protein/lipids. However the MST of highest protein/fluid intake is the largest, the other direction. Therefore, this result should not be attributed to chance but to the process of selecting the nodes of the subnetworks. In this case, protein/lipid consumption. If data

	size were large enough then we could perform this gedankenexperiment and show statistically this theoretical argument. As this data set size is really small it is not possible. Nevertheless the reviewer idea is very good, and could be implemented in other datasets. In that sense we are aware that there might be limitations related to this small size for using more rigorous statistical treatment. On the other hand literature is pointing to the importance of doing microbiota studies in nonwestern populations, and minorities are usually under-represented in mainstream literature. Not just because they represent a very interesting contrasting population, but also to fight against dominant culture bias. This translates into unavoidable population size problems.<br><br>As the reviewer suggested, we did a test for distribution because data suggested a non-normal dist. Nevertheless, that was not the case for either protein or lipid. We performed a Shapiro test and our data resulted normally distributed (For Protein; W = 0.94715, p-value = 0.1302. For Lipids; W = 0.96779, p-value = 0.4604). Given these results, we totally agree with the reviewer that it could be more robust to divide in three categories by percentiles. However, this approximation makes each subgroup have a very small sample size.<br>With this in mind, our strategy of considering the mean to divide population resulted in two statistically different groups of consumption. We did a T-test for lipids and proteins, and in both cases the mean threshold value for defining Low and High produce statistically different groups. This was clarified in the manuscript.In addition, we included an additional paragraph that justify the use of categories of consumption in order to construct comparative networks in GM and BCA .
For the GM network, the author first selected the ASVs based on random forest to classify high/low intake group. I think this step introduced bias when build GM	This is an essentially confusing explanation in the methods section. Thanks to the reviewer for noticing it. We revised and corrected it. We did use the complete ASV data set. The

network. This network may not represent the full gut microbiota (only 700+ out of 1900+ ASVs are selected). I think the better way is to build a network based on all ASVs or the high abundant/prevalent ASVs to get the full picture of the gut microbiota.	random forest analysis was an exploratory exercise to identify if the effect of protein and lipid intake differences in GM is systemic or not ,it is.
The method section of "Network analysis for EEG and GM data" is not clear. a, the protein/lipid intake group part is belonged to "Protein and Lipids intake estimation". b, stepwise details are needed for building brain power network and gut microbiota network. c, the introduction of MST should be in the beginning or in the end, not in between of brain power network and gut microbiota network, and the pseudo code is not necessary. d, a flowchart may help understand the steps.	We revised the section, improved and include a data analysis basic flow diagram (now Figure 2)
The format of citation is not consistent along the manuscript. Most of the citations are in parentheses, but a few are in superscript. like reference 20 in page 3, 33 in page 5, 42 in page 6, 54, 55 in page 13.	Thank you, we have included the missing citations. Additionally, We changed all references to bibtex and used the PLoS-ONE bst file. This should ensure that citation along the MS is consistent and the format in the reference list should be too.
Page 4, study site and participants, the number of children is 33, but "12 males and 19 females".	Revised, the correct number of participants is 31, and it is now corrected throughout the document, thanks for noticing this.
What is the correlation between protein and lipid intake?	We need clarification as to what correlation is referred to. For example, if the question refers to whether we found a correlation between lipids and protein consumption, we can say that this correlation is high. However, some foods contained more of one or the other nutrient and were consumed in different amounts by the participants. These variations explain why we found different sample sizes in the high and low categorized groups between the two nutrients. Added to this, we also found that the distribution of consumption of these nutrients was different. However, we would appreciate a more specific question to respond satisfactorily to this concern.

The citation for R Package is not a link of pdf file. Please use citation("package name") in R to get the correct citations. Page 6, 7.	Thank you for point this out, it is now corrected
In Figure 3, difference percentage in brain power networks is using "low" group as reference. But in figure 5, the difference is calculated on "high" as reference. Suggest using one group as reference in the two figures.	In figure 3 (now Figure 4) the difference is calculated High - Low. In Figure 4 (now Figure 5) High was considered 100%, and Low the corresponding %, so they cannot be put in the same way and as we see it may be confusing, we improved the figure 4 (now figure 5) to show more clearly the % of change
Have you rarefied the ASV table for gut microbiota data?	We appreciate this essential observation; Yes, plastid-encoded 16S rRNA amplicon sequence variants (ASVs) were rarefied to a minimum sequencing effort of 21,000 reads per sample. We now included this information in the manuscript.
Page 7, "With these families identified, using the dataset of ASVs (i.e. bacteria species) relative abundances in fecal samples, we used the Cooccur package (https://cran.r-project.org/web/packages/cooccur/cooccur.pdf) in R74 to construct a co-occurrence matrix and use it as weight in microbiota network". It's ASVs you identified, not "families". ASVs are not "bacteria species". And what is "R74"?	Thank you for this accurate note. ASV is not a species. We have clarified this in the "GM taxonomic assignment" section and have modified the text pointed out by the reviewer. Please see the Network analysis for EEG and GM data section.<br>In addition, R74 is a typo, should be R .Thanks fo this observation. Now is corrected
Page 12, "no Western diet", should be non-western diet.	Thank you, we corrected it and look for any other similar error in te MS
Reviewer #2	
Page 3 of 16: Correct the format of citation number 20 in the text.	We changed all references to BibTeX and used the the PLoS bst file
Page 5 of 16: In the "Gut microbiota composition" section, correct to 16S rRNA instead of rDNA.	Thank you, we corrected it and look for any other similar error in te MS
Page 6 of 16: In the "Network analysis for EEG and GM data" section, put spaces after the "=" sign and adjust throughout the document.	Thank you for the suggestion, we corrected it and look for any other similar error in te MS

Page 6 of 16: Remove "the" in (the 31 children).	Corrected
In methods, mentioned 33 participants and in results 31 children.	Revised, the correct number of participants is 31, and it is now corrected throughout the document, thanks for noticing this.
Page 6 of 16: Figure 1. The figure must have a title and then, the explanation that is included. Please indicate the sample size as n = 9, n = 22, n = 11 and n = 20 and not just the isolated value.	Thank you for pointing this out, we corrected it as suggested.
Page 6 of 16: Correct the format of citation number 42 in the text.	We changed all references to BibTeX and used the the PLoS bst file
Page 7of 16: Change in the text "study samples" to "study participants". Also adjust the title of Table 1 in the same way.	Thanks for the suggestion, we changed it in accordance.
Page 7 of 16: Simplify the format of Table 1, placing the value and its deviation for males in a single column and in another for females (not in separate cells). Indicate in the header if it is mean or median and if it is standard error or standard deviation. Indicate in the title of the table that it is "according to sex". It is recommended to add a column with the value of p and in the table footer indicate the realized statistical analysis (for example, t-test for independent samples).	Thank you for pointing this out, we add in the table footer that we are reporting mean +/- standard deviation.<br><br>Using a Wilcoxon-Mann–Whitney test we found no significant difference for any variable in Table 1 in terms of sex. As we are not using sex in analysis in the MS we did not included the test results. We now improved table title and footer to include this information.<br><br>Age:<br>U=106; P=0.74<br><br>Height:<br>U=101.5; P=0.61<br><br>Weight:<br>U=97; P=0.49<br><br>Head circumference:<br>U=94.5; P=0.42<br><br>Brachial perimeter:<br>U=73; p=0.13<br><br><br>Protein intake:<br>U=111.5, p=0.919<br>Lipid intake:

	U=113.5, p=0.983
Page 8 of 16: Table 2. It must have a title and table footer. Unify format, avoid writing in capital letters, indicate if it is mean or median and if it is standard error or standard deviation.	Capitalization corrected. We include in table footer we are reporting mean and standard deviation
Page 9 of 16: Figure 2. Please include a figure title before the explanation.	Thank you we revised the document for similar issues and corrected it
Page 11 of 16: Figure 4, unify 4a and 4b in lowercase as mentioned in the text, instead of A and B. It is recommended to cut the x-axis, for example up to 1000.	Thank you for the valuable suggestion, the figure did improve because of it.
age 11 of 16: "Finally, anthropometric and dietary patterns were not different between sex or age (Table 1). Hence, we did not include any of these variables as predictors of brain cortex or GM connectivity." The authors do not present a statistical analysis showing that there are no differences. It is recommended to include it as part of the table.	Thank you for noticing. We think that the line may be misleading and we delete it.<br><br>Test results for table 1 were included in the response to prior comment.
Page 13 of 16: In paragraph 2: "and in which essential factors,as diet, might profoundly influence the gut-brain signaling in adulthood (4).", include a space and the word "such" before "as diet ".	Thank you, we corrected it.
Page 13 of 16: Correct the format of citations number 54 and 55 in the text.	Thank you for noticing this, we corrected it.
All participants are between 5-10 years old. Please adjust the Informed Consent Statement, removing the adult participants.	Thanks for this observation. Because participants do not reach the age of majority, the parents or caregivers signed the consent statement. This protocol follows the ethical protocol approved for this study. (FPSI/CE/01/2016-UNAM). We have now clarified this in the manuscript in the section of " **Procedures to recruit participants.**
