# Supplementary material for: Similar connectivity of gut microbiota and brain activity networks is mediated by animal protein and lipid intake in children from a Mexican indigenous population
Source: PLoS One. 2023 Jun 29;18(6):e0281385. doi: 10.1371/journal.pone.0281385 (PMC10310019; doi:10.1371/journal.pone.0281385)
Supplement: Attachment — Submitted filename: Response Letter Minor Rev_Jan11.pdf [file pone.0281385.s006.pdf]

Response to Reviewers
Second rev. for PONE-D-22-17440

Similar connectivity of gut microbiota and brain activity networks is mediated by animal protein and lipid intake in children from a Mexican indigenous population.

We are thankful for useful comments by reviewers that have improved the quality of the work. Here we track how we took editor and reviewers comments into account**.**

PLoS comments	
Comment	Response
Journal Requirements:<br><br>Please review your reference list to ensure that it is complete and correct. If you have cited papers that have been retracted, please include the rationale for doing so in the manuscript text, or remove these references and replace them with relevant current references. Any changes to the reference list should be mentioned in the rebuttal letter that accompanies your revised manuscript. If you need to cite a retracted article, indicate the article's retracted status in the References list and also include a citation and full reference for the retraction notice.	We have review the references list and made some corrections:<br><br>Ref 19: Telesford QK, Joyce KE, Hayasaka S, Burdette JH, Laurienti PJ. The ubiquity of small-world networks. Brain connectivity. 2011;1(5):367–375.<br><br>Ref 32: de México y Centroamerica W. Producción de Jamaica en la montaña alta de Guerrero, proyecto apoyado por Fundación Walmart;. Available from: https://www.youtube.com/watch?v=WOEcGUHjR9Q.<br><br>Ref 46: Pardy C. Mixed-Pair Mutual Information Estimators; 2022. Available from: https://r-forge.r-project.org/projects/mpmi/.<br><br>Ref 50:Liaw A, Wiener M. Classification and Regression by randomForest; 2002. Available from: https://CRAN.R-project.org/doc/Rnews/.<br><br>Ref 51: Griffith DM, Veech JA, Marsh CJ. cooccur: Probabilistic Species

	Co-Occurrence<br>Analysis in R; 2016. Available from: https://cran.r-project.org/web/packages/cooccur/cooccur.pdf.<br><br>References after reference 32 were moved forward because this reference was added. Happening the same after adding references 46, 50 and 51.
Reviewer #1	
I think the authors answered most of my previous questions, although it takes a lot of time to check out every point since they didn't mention the exact location in their revised manuscript to each question.	We apologize for this and the subsequent inconvenience, we are now mentioning the exact location of all changes, in green.
I still have the question regarding Lipid intake and protein intake. How many subjects belong to high lipid intake AND high protein intake? How many subjects belong to low lipid intake AND low protein intake? I think it's important to know the proportion of overlapped subjects in high lipid and high protein group or low lipid and low protein group.	There are 11 individuals with high protein intake, 9 with high lipid intake, and 6 individuals that are both in the high protein and lipid intake, corresponding to 42.8% shared. On the other hand, there are 20 individuals with low protein intake, 22 with low protein intake, and 16 both with low protein and lipid intake, corresponding to 61.5% shared.<br><br>We included this test in lines 160-164
In their response they explained that their dataset is small and is not possible to have some statistic test on the MST difference between high/low nutrition intake group. I think it's very important that they should also mention this limitation in their manuscript.	We agree with reviewer, and have clearly state it at the end of the discussion section (lines 386-412):<br><br>As we have discussed above, current research in the field is pointing to the importance of doing microbiota studies in nonwestern populations, and how minorities are usually under-represented in mainstream literature. This is relevant not just because they represent very interesting contrasting populations, but also to fight against dominant culture bias. Nevertheless, due to other limitations such as lack of funding, difficulties in access, or just because those populations are themselves small;

studying them may potentially translate into unavoidable sample size problems.

This is the case of our work, which has a too small sample size to do a more rigorous statistical treatment on the MST difference between high/low nutrition intake groups. An issue was highlighted by an anonymous reviewer during the peer review process. In particular, it would be desirable to randomly construct k subnets of sizes n, m (with $n<m$); and then compare pairs of subnets of n nodes vs. subnets of m nodes; to statistically respond: How the difference of weight of the MST would be if randomly divide the data into two groups?

Although the sample size is not large enough to perform this gedankenexperiment we offer a theoretical argument.

Let's first consider that If there were no underlying ecological (external to the individual) population process, the mutual information matrix (MI) would be very homogeneous. These coefficients of the MI matrix are used as the weight of the networks, so weights are also very homogeneous. It would be expected then, that the value of the MST would change mainly in the function of the size of the network. This would mean that the MST of networks of m nodes would be greater than the MST of n nodes.

In our case study, the subnetwork with the highest number of nodes corresponds to the lowest intake of protein/lipids. However, the MST of the highest protein/fluid intake is the largest, in the other direction. Therefore, this result should not be attributed to chance.

The author claimed they have corrected the format for R package citation, but they didn't! line 241 and 249 are still two hyperlinks. I found the instruction for citation of these two packages below:<br><br>cooccur citation info (r-project.org), randomForest citation info (r-project.org).<br><br>What they need is just use the BibTeX entry from those links as the correct reference.	Sorry for this, we have now taken care of it and now it is found on lines 244 and 250-251 as following:<br><br>"...implemented in R under the randomForest package [50], and we identified essentia"<br><br>"... for this reason, we used the complete ASVs data set as input to a co-occurrence analysis using Cooccur package in R [51]."<br><br>Also corrected citation on line 226:<br><br>"Mutual Information (MI) matrix using the 'mpmi' R Package [46]."
They now merged mean decrease gini of protein and lipid together in fig 5. That figure maybe misleading because in x axis the ASVs are arranges as the gini importance value decrease. When merge the two panels, it gives the reader the impression that the gini importance value ranks the same for the ASVs in protein intake and lipid intake. But I don't think that's true.	Thank you for this detailed review. We really appreciate all your work. We modified the figure to take care of this comment by separating into two panels
